# Self-Perceived Instructional Competence, Self-Efficacy and Burnout during the Covid-19 Pandemic: A Study of a Group of Italian School Teachers

**DOI:** 10.3390/ejihpe11020035

**Published:** 2021-06-01

**Authors:** Monica Pellerone

**Affiliations:** Faculty of Human and Social Sciences, “Kore” University of Enna, CAP 94100 Enna, EN, Italy; monica.pellerone@unikore.it; Tel.: +39-3294324311

**Keywords:** instructional competence, educational well-being, self-efficacy, learning process, teacher

## Abstract

Prolonged school closures, forced isolation, and mutations in social interactions due to the COVID-19 pandemic have posed challenges for actors in the educational context; teachers, in particular, have had to develop new instructional strategies to ensure that lessons could continue. The present research measures in a group of 374 Italian teachers—curricular and specialist support teachers—the relationship between self-perceived instructional competence, self-efficacy, and burnout. The present research, conducted between April and December 2020, represents the second part of a larger study conducted from November 2018 to October 2019, which was replicated during COVID-19. Participants completed an anamnestic questionnaire, the Assessment Teaching Scale, and the Maslach Burnout Inventory in both phases of research; an ad hoc questionnaire (to measure teaching practices) and the Teacher Sense of Self Efficacy Scale were added in the second phase. Data confirm that general level of burnout increased and personal accomplishment was reduced during the pandemic; elevated personal accomplishment appears to be a predictor of emotional, socio-relational, and didactic competences before and during the pandemic. Feelings of frustration and accomplishment represent some manifestations of distress caused by the pandemic condition; these dynamics favor the crystallization of roles and behaviors towards the perception of metacognitive teaching processes.

## 1. Introduction

One of the factors involved in the teaching process is the adequacy of the instructional strategies, which allows the students to acquire knowledge and a positive connection within the class group [1]. This adequacy not only depends on the teachers’ curriculum, but it also depends on their transversal skills, such as knowledge, method, ability of communication, relationship, evaluation, and sensitivity for the social context [2].

The literature [3] highlights how an adequate teaching–learning process must have three main aspects, namely, possessing a cognitive component (the construction of meaning), an affective component (the attribution of meaning), and carrying out a social function. In this perspective, teachers must know how to establish emotional relationships with students and create a positive classroom atmosphere, showing interest in their learners, trust in their potential, and a desire to transmit knowledge. Furthermore, teachers must be witnesses of values, interested in the social formation of students, and open to the change and reflection that comes from them.

Some authors suggest that perceived instructional self-efficacy—defined as a judgment of the teacher’s capabilities to bring about desired outcomes of student engagement and learning—can affect an important part of didactic efficacy [4], although the teacher’s perceived self-efficacy is strongly influenced by their ideal image and to a lesser extent by objective evaluations of their work [4,5,6,7,8].

Even relational variables, which fall within transversal skills, influence the instructional competence and ways of managing and directing the class, which, if being flexible, will adapt to demands and contingencies of the class group. Transversal skills, in particular, appear to be fundamental requirements to face situations of great change, such as the COVID-19 pandemic, which hit the whole world starting in March 2020.

COVID-19 (acronym for coronavirus disease 2019) represents, in fact, a challenge on multiple fronts: health, economic, social, and educational. With reference to the Italian educational context, starting in March 2020, the government ordered the temporary closure of schools throughout the nation as a measure to prevent the spread of the infection. Furthermore, the high number of asymptomatic subjects, presenting the same viremia and high transmissibility, made it necessary to implement measures of containment and social distancing [9,10]. Italian schools, in accordance with the provisions of the government, implemented remote teaching systems to allow students and teachers to complete the school year, through synchronous (real-time lessons) and asynchronous lectures (the storage and dissemination of educational material, such as video lessons, handouts, audio, and films). In particular, the government provided funds to equip schools with digital tools and for the use of e-learning platforms, which were scarcely present in Italian schools. Furthermore, in order to help less well-off students, the government provided for the free loan of individual digital devices and the online training of teachers on distance teaching methodologies and techniques.

Although distance learning—compared to normal classroom-taught lessons—is characterized by a greater flexibility in programming and the ability to customize learning processes and easy distribution of information [11,12,13], it can present a barrier for younger students [14]. In particular, distance learning requires the student to be able to self-monitor and regulate their motivation to learn [15,16,17], determining a higher risk of passive procrastination in younger students or dropouts in older students. For example, a recent Indian study found that in the use of virtual classrooms, the average of the actual benefits was significantly lower than the average of the expected benefits. The main problems of virtual classrooms seemed to be network problems, lack of training, and reduced interaction due to connection problems [18].

In addition, Xie and Yang [19], in a study on autonomous learning during the pandemic, found two main critical issues: socio-economic differences and the lack of targeted autonomous learning materials. Regarding the first point, it emerged that not all students had electronic devices (phones, tablets, computers), the connection signal was poor or insufficient, and there were conflicts within the family. With regard to the second element, in classroom teaching, the teacher designs the teaching process based on the learning objectives of each lesson, identifying the connection between old and new knowledge and developing students’ ability to cooperate in groups; with online teaching, due to limited interaction, students have to study independently by exploring textbooks and materials provided.

Furthermore, Kong [20] observed that primary school students could not study effectively at home due to various problems, such as fundamental changes in the study environment (transition from a traditional classroom to online learning), but above all unsatisfactory learning because of reduced supervision by the teachers and parents. The author also pointed out that teachers have difficulties in adapting to the new online mode: They cannot express themselves in front of a webcam, their language lacks flexibility, the form is simple and does not attract students, and they do not interact in real time with students, leading to a low level of participation.

The pandemic situation, therefore, has posed great challenges for all actors in the educational context. Teachers in particular have had to develop new instructional strategies to ensure that lessons can continue without interruption [21]. Parents, on the other hand, took on the role of teachers in addition to their work and family obligations. Students also found themselves in a new situation: Whereas in classroom-taught lessons, fixed structures regulated daily school life and learning time, students now had to organize and regulate their learning independently [22].

### 1.1. Teachers’ Self-Efficacy and Burnout

Prolonged school closures and confinement at home have had negative effects on the mental and physical health of children and adults [23]. In teachers in particular, psychological problems strictly related to home isolation are added to pre-existing pictures of burnout, a syndrome resulting from chronic stress in the workplace; this appears to be characterized by a feeling of depletion of energy or exhaustion, an increase in mental distance and negative or cynical feelings towards work and others, and reduced professional efficacy [24,25].

The literature prior to the pandemic shows how part of teachers perceived their work as very stressful due to specific elements, such as the presence of an excessive workload, lack of resources, poor feedback received from students, frequent lack of support from colleagues and the head teacher, wage inadequacy, and an often problematic relationship with parents [26]; to these are added low self-esteem and control over one’s work, reduced knowledge of work goals, and difficulties in managing extra-didactic situations [27]. The consequences of burnout on teachers are generally characterized by and increase in levels of absenteeism, substitution, and early retirement, and a deterioration in instructional self-efficacy.

The literature shows how their levels of burnout seem to increase in relation to some independent variables, such as age, the grade of school they teach, and level of experience [28], but above all instructional effectiveness and perception of self-efficacy. In fact, the well-being and psycho-physical health of teachers appears to be closely correlated with perceived self-efficacy in teaching, good performance, and didactic–metacognitive strategies [29,30]. In particular, teachers with a lower level of burnout and a strong sense of instructional effectiveness, planning, and organizing are more open to new ideas and more willing to experiment with new methods of improving and meeting the needs of their students [31]. Furthermore, they are likely to exert a positive influence on student achievement and their sense of effectiveness [32].

On the other hand, Skaalvik and Skaalvik [33] showed that job satisfaction is positively correlated with self-efficacy, and that emotional stress represents an important predictor of the perception of didactic self-efficacy. This is probably due to the fact that as teachers with low burnout levels become more aware of their ability to teach, their self-efficacy increases; consequently, their self-competence improves because they develop a metacognitive and self-regulated approach through a reflection on why, what, and how to teach [34].

More recently, Pellerone et al. [35] underlined the predictive role of emotional stress, depersonalization, and self-fulfillment on the didactic quality of primary school teachers. In particular, the authors showed how teachers with high levels of stress more frequently use negative coping strategies such as avoidance; on the contrary, teachers with elevated experience and personal fulfillment seem to have higher emotional, socio-relational, and didactic skills. In such circumstances, the socio-emotional characteristics of teachers seem to be an important mediator of the relationship between their emotional stress and didactic strategies.

In the light of the literature examined, it would seem interesting to analyze the changes that have occurred in teachers’ condition during the COVID-19 pandemic, and in particular the possible mutations in the effectiveness of teaching processes and in the educational strategies adopted by teachers, following a modification of the levels of burnout.

### 1.2. Self-Perceived Instructional Competence and the Effects of Pandemic: Teachers’ Attitudes toward Change

The forced isolation due to lockdown and quarantine, the change in the context of work, and the mutation in social interactions and other factors related to the pandemic could have worsened the levels of burnout or caused its onset. Although the literature is not yet exhaustive in this regard, a recent study by Cardullo et al. [36] highlighted how in the pandemic phase, among the difficulties encountered by teachers were the lack of technological devices and the low level of perceived self-efficacy in the use of technology, the lack of parental support in on-line learning, and above all, the difficulty in motivating and involving students in remote contexts. On the other hand, many teachers affirmed the usefulness of online teaching in small subgroups thanks to the use of additional resources, and they also mentioned less peer pressure and fewer distractions in these contexts.

However, despite the benefits of technology, we cannot underestimate some mediating factors that often hinder the online learning environment, such as teachers’ confidence in their ability to face new challenges through flexible teaching styles and new educational strategies: Many teachers feel overwhelmed, unprepared, and mentally exhausted as they try to successfully navigate the online learning environment. In this regard, Pressley [37] showed how for teachers who returned to class during the COVID-19 pandemic, stressors more proximal to burnout were COVID-19-related anxiety, anxiety about teaching requests, reduced communication with parents, and lack of administrative support. The author also demonstrated how levels of burnout did not differ significantly according to ethnicity, the geographical location in which the school was located, the teaching experience, and the teacher’s education level.

In addition, Ibanez et al. [38] showed the presence of higher levels of burnout in female and older teachers: This difference was mediated by the lack of knowledge of computer science. In fact, the authors showed that the computer skills possessed by women appear to be lower than those of men; this determined, in the group of women, a greater probability of expressing negative emotions and a high workload. An age difference related to digital skills was also detected during the pandemic, as the progressive widening of the age gap has a major impact on student education in a world where technology has taken on special relevance.

A longitudinal study conducted by Sokal et al. at the beginning of the pandemic [39] showed how the effectiveness of the teacher, the attitude towards change, and the perception of support were correlated with resilience and burnout. In particular, during the first three months of the pandemic, teachers showed increasing exhaustion and cynicism, but also greater effectiveness for classroom management and a sense of achievement. The same authors, confirming the previous literature [40], suggested that the intensity and nature of a challenge depended on both the context and the personal interpretation; in particular, in the teaching context during COVID-19, the lack of resources (such as support from parents or professional learning methods) was negatively correlated with achievement, showing that if the perception of reduced resources increases, the perception of results decreases.

It is therefore essential to analyze these barriers in order to provide the resources that allow teachers to move from expected behavior to actual behavior by acting on three components, namely, cognitive, affective, and behavioral intention [41,42,43].

### 1.3. Objective and Research Hypotheses

Within a metacognitive approach, the present research examines the variables inherent in the teacher as an agent, taking into account the dual nature of their dynamic activity: a more individual one (i.e., the emotional component) and a more social one (i.e., the socio-relational and organizational components).

The purpose of this research is to measure the self-perceived instructional competence, self-efficacy, and burnout in a group of Italian teachers, and curricular and specialist support, comparing these dimensions before and during the pandemic; to value the predictive role of the possible presence of burnout on the quality of teaching; and to analyze the mediating role of teachers’ self-efficacy on the relationship between burnout and self-perceived instructional competence.

In particular, the following hypotheses were formulated:

**Hypothesis** **1** **(H1).** *The socio-emotional factor (composed of variables related to relationship skills and inner balance) and the didactic factor (composed by skills of adaptation to new situations, planning, and didactic control) were reduced during the pandemic.*

**Hypothesis** **2** **(H2).** *The burnout level—in particular, personal accomplishment—was reduced during the pandemic.*

**Hypothesis** **3** **(H3).** *Personal accomplishment is a predictive variable of emotional, socio-relational, and didactic abilities before and during the pandemic.*

**Hypothesis** **4** **(H4).** *Before the pandemic, socio-emotional, communicative-relational, and didactic abilities were predicted by the years of experience, presence of disabled students, and number of students per class, and during the pandemic only by the years of experience.*

**Hypothesis** **5** **(H5).** *Teachers’ self-efficacy was a mediator of the relationship between burnout and self-perceived instructional competence during the pandemic.*

## 2. Materials and Methods

The present research was conducted between April and December 2020, during the COVID-19 pandemic. It represents the second part of a larger study that was conducted from November 2018 to October 2019 (Time 1), which was replicated during COVID-19 (Time 2).

In the first phase (Time 1), the research involved 374 Sicilian teachers, aged between 20 and 66 years old (M = 45.78; SD = 10.16), who worked in three school orders: kindergarten, primary, and middle school. In the second phase of the study (Time 2), 344 teachers were traced (mean age of 46.92, standard deviation of 10.40, and range between 22 and 68 years), with a mortality rate of 8%.

Inclusion criteria comprised the desire and satisfaction of participating in the research.

Consent of the school authorities was sought before the distribution and collection of the instruments. The questionnaires were anonymous and the participants were given the following information: the purpose and structure of the study, voluntary participation, a guarantee of anonymity, and the free will to withdraw from participation, with no disadvantage upon withdrawal. All participants provided written informed consent.

The Internal Review Board (IRB) of the Faculty of Human and Social Sciences at the University of Enna “Kore” approved the administered instruments and the research project.

The instruments were administered by qualified researchers, and teachers were given 50–60 min to complete them. The participants were from a medium to high socio-economic background.

Participants completed an anamnestic questionnaire, the Assessment Teaching Scale (ECAD-EP), and the Maslach Burnout Inventory (MBI) in both phases of research; in the second phase only, an ad hoc questionnaire that analyzed teaching practices during the pandemic and the Teacher Sense of Self Efficacy Scale (TSES) were completed.

Anamnestic data were collected through the administration of a questionnaire divided into two parts: the first to acquire basic information, age, sex, years of experience, and role at the school (curricular or specialist support teacher), and the second to establish the number of students per class, presence of students with disabilities, and grade they teach.

The ECAD-EP questionnaire, or Escala de Evaluación de la Competencia Autopercibida of the Teacher of Primary Education [44], stems from the need to create a valid and accurate tool able to identify and thus being able to measure the strategic competences of schoolteachers. This is a questionnaire on a 5-point Likert scale (from “strongly disagree” to “strongly agree”) consisting of 58 items grouped into three factor dimensions:

- Socio-emotional factor (Factor A), which measures the ability to mediate and involve the class group, adaptability, communicative sensitivity, the capacity to establish a healthy coexistence, affective involvement, empathy, and self-efficacy;
- Communicative-relational factor (Factor B), which includes assertiveness, affective and executive leadership, conflict resolution, and nonverbal and para-verbal communication;
- Didactic factor (Factor C), which is linked to the management processes of teaching; it refers to skills of adaptation to new situations, planning, and didactic control.

The literature and the present study report sufficient estimates of the internal validity of the tool (Table 1).

The Maslach Burnout Inventory is a 22-item questionnaire on 7-point Likert scale (from “never” to “every day”) designed to assess the level of burnout [45]. It is a multidimensional questionnaire that addresses three areas of expertise: emotional exhaustion, depersonalization, and personal accomplishment. In particular, emotional exhaustion constitutes the answer to a working situation that induces excessive emotional involvement and emotional overload. Depersonalization is manifested by a detached, sometimes decidedly negative and hostile attitude towards users. Reduced personal accomplishment is substantiated in an exhausting feeling of inadequacy to establish an effective relationship of help with its users and implies a reduced level of self-esteem and the attenuation of the desire for success.

The Cronbach’s alpha coefficient was equal to 0.87 for the emotional exhaustion scale, 0.71 for the depersonalization scale, and 0.78 for the personal accomplishment scale [46]. In the present study, the Cronbach’s alpha coefficient was equal to 0.85 for the emotional exhaustion scale, 0.67 for the depersonalization scale, and 0.71 for the personal accomplishment scale in the first phase, and 0.87 for the emotional exhaustion scale, 0.69 for the depersonalization scale, and 0.72 for the personal accomplishment scale in the second phase.

The ad hoc questionnaire measured teaching practices during two last months related to the pandemic condition. It is a questionnaire on a 5-point Likert scale (from “strongly disagree” to “strongly agree”), consisting of 12 items grouped into three factors: emotional, didactic, and relational dimensions. Some examples of items are the following:

- “I am aware that my task is also to accompany and support the students” (emotional dimension).
- “I use the electronic register as my first organizational channel, but I don’t limit myself to it” (didactic dimension).
- “I am aware that in an emergency situation the priority is not to lose the continuity of relationships with students” (relational dimension).

Teaching efficacy was measured using the Teacher Sense of Self-Efficacy Scale [47]. This measure includes 12 questions related to three aspects of efficacy—efficacy in student engagement, efficacy in instructional strategies, and efficacy in classroom management—and each question is on a Likert scale from 1 (not at all) to 9 (a great deal).

The literature and the present study reported good estimates of internal validity of the tool. The Cronbach’s alpha coefficient was equal to 0.87 for the engagement scale, 0.91 for the instruction scale, 0.90 for the management scale, and 0.90 for the general level of self-efficacy [47]. In the present study, the Cronbach’s alpha coefficient was equal to 0.85 for the engagement scale, 0.87 for the instructional scale, 0.91 for the management scale, and 0.90 for the general level.

## 3. Data Analysis

All analyses were conducted with Statistical Package for the Social Sciences 26.0 (IBM Corporation, Armonk, NY, USA). The level of significance was 0.05.

Descriptive analysis was used to assess the mean scores of all variables.

In reference to the Assessment Teaching Scale, the multivariate analysis of variance (MANOVA) was used to measure the influence of independent variables (age, years of experience, type of function, school grade, presence or absence of students with disabilities, and number of students per class) in ECAD-EP subdimensions. In reference to the burnout level, the multivariate analysis of variance (MANOVA) was carried out to value the potential effect of the independent variables on MBI subdimensions. The same analysis was carried out to measure the influence of independent variable on teaching practices and self-efficacy during the pandemic.

To compare the level of burnout and the self-perceived instructional competence before and during the COVID-19 pandemic, a *T* test for an independent group was conducted; the hypotheses was tested using Bonferroni’s correction of significance level.

The hierarchical regression for separate blocks was used in order to explore the predictive contribution of the burnout on the self-perceived instructional competence at Time 1 and Time 2, including anamnestic data (in the first block) and the MBI subdimensions (in the second block).

Furthermore, a mediation analysis was conducted in order to identify whether the relationship between burnout and self-perceived instructional competence was mediated by teachers’ self-efficacy during the COVID-19 pandemic.

## 4. Results

### 4.1. Inferential Analysis

A descriptive analysis was conducted in order to investigate the anamnestic data. In reference to the school order, 67.4% of participants worked in a primary school, 26.6% teaches in a middle school, and 6% in kindergarten. The participating teachers in the research had an average experience of 21.60 years (with a range between 2 and 44 years and standard deviation equal to 11.47); in reference to their role, 78.8% were curricular teachers, and 21.2% were specialist support teachers. The average number of students for each classroom was equal to 21.64 (with a range between 6 and 30 students). Furthermore, 83.4% declared to have students with an intellectual and/or physical disability, and 16.6% declared to not have students with a difficulty.

In reference to the ECAD-EP scores, the MANOVA was carried out in order to verify the influence of personal variables (age, years of experience, and role of teachers) on socio-emotional, communicative-relational, and didactic factors at Time 1 and Time 2.

In reference to the emotional factor at Time 1, the multivariate analysis of variance (MANOVA) showed how years of experience seemed to influence the ability to mediate (F = 1.98; *p* < 0.01) and self-efficacy (F = 1.89; *p* < 0.01). Tukey’s post hoc underlined that teachers with an elevated experience tended to manifest a higher level of mediation and self-efficacy. At Time 2, only mediation was influenced by experience (F = 1.63; *p* < 0.05).

In reference to the communicative-relational factor at Time 1, nonverbal communication seemed to be influenced by experience (F = 1.92, *p* < 0.01), and affective leadership by role (F = 4.95, *p* < 0.05). Tukey’s post hoc underlined that teachers with more experience used nonverbal communication, but support teachers tended to manifest a lower level of affective leadership than curricular teachers. At Time 2, only nonverbal communication (F = 1.63, *p* < 0.05) was influenced by experience; age influenced conflict resolution ability (F = 1.72, *p* < 0.05) and executive (F = 1.68, *p* < 0.05) and affective leadership (F = 1.60, *p* < 0.05), which was also influenced by role (F = 5.47, *p* < 0.05). So, similar to Time 1, teachers with more experience used nonverbal communication, and older teachers presented a higher level of affective and executive leadership and an elevated ability to resolve conflict.

Regarding the didactic factor at Time 1, planning ability seemed to be influenced by age (F = 1.69, *p* < 0.05) and experience (F = 2.02, *p* < 0.01), which also influenced instructional control; moreover, interaction between age and experience seemed to influence planning ability (F = 1.60; *p* < 0.05) and situational adaptability (F = 1.44; *p* < 0.05). Interaction between experience and role seemed to influence the perceived instructional control (F = 2.89; *p* < 0.01). In particular, as underlined by Tukey’s post hoc analysis, older teachers and/or those with more experience presented elevated planning ability and adaptation to new situations; teachers with more experience—above all, curricular teachers—perceived reduced instructional control. At Time 2, instructional control was influenced by age (F = 2.08, *p* < 0.01) and experience (F = 1.69, *p* < 0.05), which also influenced planning ability (F = 2.11, *p* < 0.05); the interaction between experience and role seemed to influence instructional control (F = 4.29; *p* < 0.05). In detail, Tukey’s post hoc analysis underlined that younger teachers or those with less experience perceived elevated instructional control that seemed to be reduced in the curricular teachers with more experience.

In reference to the ECAD-EP, the same analysis was carried out in order to verify the influence of organizational variables (number of students, the presence or absence of students with disabilities, grade taught) on the socio-emotional, communicative-relational, and didactic factors. In reference to the emotional factor at Time 1, the data analysis underlined how grade influenced communication sensibility (F = 3.16, *p* < 0.05), mediation (F = 6.29, *p* < 0.001), group dynamization (F = 4.06, *p* < 0.01), and self-efficacy (F = 7.08 *p* < 0.001). The interaction between the number of students per class and disabled students influenced the level of self-efficacy (F = 2.24, *p* < 0.01), which was also influenced by the interaction between the presence of disabled students and grade (F = 6.74, *p* < 0.001). Tukey’s post hoc underlined that the kindergarten teachers used a higher level of communication sensibility, but those in primary school had an elevated mediation ability; in the middle school they manifested a reduced use of group dynamization and level of self-efficacy. At Time 2, only the grade seemed to influence the use of mediation instrument (F = 4.76, *p* < 0.01), which appeared more present in the primary school.

In reference to the communicative-relational factor at Time 1, nonverbal communication (F = 4.24, *p* < 0.01), conflict resolution ability (F = 2.97, *p* < 0.05), and affective (F = 2.95, *p* < 0.05) and executive leadership (F = 3.04, *p* < 0.05) seemed to be influenced by the grade in which the teachers worked. Tukey’s post hoc underlined that in the kindergarten, teachers used more nonverbal communication and affective leadership; in the middle school, they manifested elevated executive leadership and problem-solving abilities. Furthermore, MANOVA underlined how the interaction between the presence of disabled students and number of students per class influenced the reduced use of affective leadership (F = 1.19; *p* < 0.05). At Time 2, teachers who worked in the kindergarten used pre-verbal communication (F = 3.97, *p* < 0.05).

Regarding the didactic factor at Time 1, the grade only seemed to influence the perception of instructional control (F = 2.78, *p* < 0.05) and planning ability (F = 2.68, *p* < 0.05): In particular, in the middle school, teachers perceived a reduced level of instructional control, but in the primary school an elevated level of planning ability. Similarly, at Time 2, the grade influenced the perception of institutional adaptation (F = 5.13, *p* < 0.01) and planning ability (F = 4.90, *p* < 0.01), which appeared to be elevated in teachers in the primary school, in opposition to the institutional adaptation that was elevated in the middle school.

The MANOVA, carried out to verify the influence of personal variables (age, years of experience, and role) on burnout subdimensions at Time 1, underlined the main effect linked to the experience on the general level of burnout (F = 1.70; *p* < 0.05): Teachers with elevated experience manifested a reduced level of burnout. At Time 2, the MANOVA emphasized the influence of the interaction effect between experience and age on personal satisfaction (F = 1.50; *p* < 0.05): In particular, younger participants with elevated experience tended to manifest great personal satisfaction.

Another MANOVA was carried out to verify the influence of independent organizational variables (number of students, presence or absence of students with disabilities, grade) on burnout subdimensions at Time 1. The analysis showed that grade influenced personal accomplishment (F = 3.36; *p* < 0.05) and depersonalization (F = 4.40; *p* < 0.01), which appeared to also be influenced by the presence of disabled students (F = 7.00; *p* < 0.01). In particular, the descriptive analysis showed that participants teaching in a lower grade school tended to manifest reduced accomplishment and depersonalization; similarly, teaching in a context with disabled students determined an elevated perception of depersonalization. At Time 2, grade influenced depersonalization (F = 5.20; *p* < 0.01) and general level of burnout (F = 3.73; *p* < 0.05); depersonalization was also influenced by the presence of disabled students in the class group (F = 1.67; *p* < 0.05). Likewise, participants who taught in the primary school tended to manifest a reduced level of depersonalization and general level of burnout; teaching in a context with disabled students caused depersonalization.

In reference to the teaching practices during the pandemic, the MANOVA underlined the main effect of age on the use of adaptive socio-emotional (F = 1.70; *p* < 0.05) and didactic strategies (F = 1.65; *p* < 0.05), and the interaction effect between age and experience on socio-emotional (F = 1.65; *p* < 0.05) and didactic strategies (F = 2.04; *p* < 0.01). The descriptive statistics underlined that younger teachers with elevated experience tended to use adaptive socio-emotional and didactic strategies during the pandemic.

The MANOVA, carried out to verify the influence of independent organizational variables, showed only the influence of grade on the use of didactic (F = 2.93, *p* < 0.05) and communicative strategies (F = 7.39; *p* < 0.001). In particular, teaching in a primary school influenced the use of practices characterized by didactic adaptive strategies and a simpler language, which captured the attention of young students in remote teaching.

In reference to the teachers’ self-efficacy during the pandemic, the MANOVA underlined that the engagement scale was influenced by interaction effect between the number of students per class and the grade (F = 1.65; *p* < 0.05), and between the number of students per class and the presence of disabled students (F = 4.12; *p* < 0.05); the instructional efficacy was influenced by the interaction between the number of students per class, disabled students, and grade (F = 3.16; *p* < 0.05). The descriptive statistics underlined that teachers working in a numerous class in the primary school or working in a numerous class with disabled students perceived a lower level of efficacy in student engagement. Teachers manifesting a lower level of efficacy in instructional strategies were those who taught in a numerous class in the primary school with disabled students.

### 4.2. Instructional Competence and Burnout during the Pre-Pandemic and Pandemic Phase

In reference to the first hypothesis, T-test analysis showed the presence of significant differences in all subdimensions of the self-perceived instructional competence except communicative sensitivity, nonverbal communication, and assertiveness (Table 2): In particular, during Time 2 (pandemic phase), the teachers seemed to manifest a lower level of instructional competence than the teachers assessed during Time 1 (pre-pandemic phase).

In reference to the second hypothesis, the same analysis underlined differences in the following subdimensions of the burnout: personal accomplishment (*p* < 0.001) and the general level of burnout (*p* < 0.001); in particular, the first subdimension seemed to be reduced (Time 1: M = 4.97, SD = 0.83; Time 2: M = 3.44, SD = 0.89) and the second seemed to be increased (Time 1: M = 2.64, SD = 0.57; Time 2: M = 2.96, SD = 0.57) during the pandemic phase.

### 4.3. Predictive Variables of Instructional Competence during the Pre-Pandemic and Pandemic Phase

In reference to the third hypothesis, multiple regression analyses were conducted to evaluate the contribution of the burnout subdimensions on the teachers’ instructional competence. In Time 1, the first regression underlined that predictors of the socio-emotional factor were, in the first step, only the years of experience (β = 0.29; *p* < 0.01) explaining 10% of the total variance, and in the second step, the level of personal accomplishment (β = 0.62, *p* < 0.001) explaining 36% of the total variance.

Similarly, the second regression analysis underlined that predictive variables of the communicative-relational factor were years of experience (β = 0.28, *p* < 0.01) in the first step (10% of total variance), and level of personal accomplishment (β = 0.39, *p* < 0.001) in the second step, explaining 27% of the total variance.

The third regression analysis underlined that predictive variables of the didactic factor were experience and role in the first step (8% of total variance), and the level of personal accomplishment in the second step, explaining 25% of the total variance (Table 3).

In reference to the fourth hypothesis, in Time 2, predictors of the socio-emotional factor were years of experience and grade in the first step, explaining 10% of the total variance, and number of disabled students, level of personal accomplishment, and emotional exhaustion in the second step, explaining 33% of the total variance (Table 4).

During Time 2, predictors of the communicative-relational factor were years of experience (β= 0.19, *p* < 0.05) in the first step (6% total variance), and level of personal accomplishment (β = 0.47, *p* < 0.001) and depersonalization (β = -0.14, *p* < 0.01) in the second step, explaining 28% of the total variance.

The third regression analysis underlined that predictive factors of the didactic dimension were the following: number of students per class and presence of disabled students in the first step (explaining 7% of the total variance), and level of depersonalization and personal accomplishment in the second step, explaining 21% of the total variance (Table 5).

### 4.4. The Mediated Effect of Teachers’ Self-Efficacy

**Figure 1:**
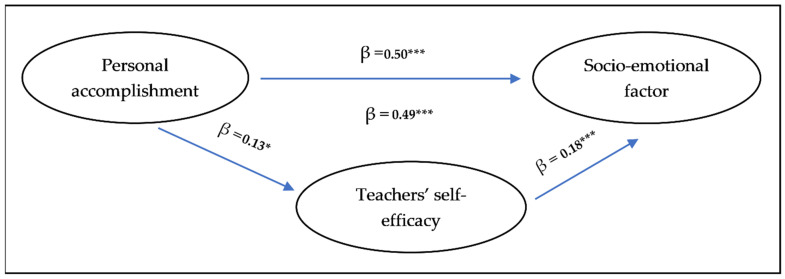
The mediated effect of teachers’ self-efficacy. * (*p* < 0.05), *** (*p* < 0.001).

In order to verify whether the teachers’ self-efficacy was a mediator of the relationship between personal accomplishment (burnout subdimension) and self-perceived instructional competence during the pandemic, another linear regression analysis was carried out (hypothesis number 5). The data showed the presence of a simple relationship between personal accomplishment and the emotional factor of ECAD-EP (β = 0.50, *p* < 0.001). Adding the hypothesized mediator, or the teachers’ self-efficacy, into the regression analysis, the beta value of the personal accomplishment became insignificant (β = 0.07, *p* > 0.05), whereas the beta value of the factor hypothesized as mediator was highly significant (β = 0.18, *p* < 0.001). Therefore, the data confirm that the level of self-efficacy can be considered a mediator of the relationship between burnout and Factor A (Figure 1).

## 5. Discussion

The objectives of the present study were the following: to measure self-perceived instructional competence, burnout, and self-efficacy in a group of Italian teachers; to value the predictive role of burnout on quality of teaching before and during the COVID-19 pandemic; and to measure the mediating role of self-efficacy on the relationship between burnout and instructional competence during the pandemic.

In the phase preceding the pandemic, the preliminary analyses show how teachers with more experience seemed to manifest greater self-efficacy, mediation, communication skills (mainly oriented to the use of non-verbal communication), planning abilities, and perception of reduced instructional control, the latter especially in curricular teachers. Support teachers, on the other hand, seemed to perceive excessive control by the head teacher and less affective leadership than curricular teachers, probably due to their precarious working condition.

Furthermore, working in a primary school led teachers to manifest an elevated ability to mediate and communicative sensitivity, which was expressed with the use of non-verbal communication, but also higher levels of planning abilities and affective leadership; this could have occurred because primary school teachers help children develop and improve their literacy skills, but above all, support students to express their emotions and behavior. On the other hand, participants who taught in the middle school tended to display great conflict resolution skills and executive leadership—suited to the adolescent phase the students were going through—although they displayed a low level of perceived self-efficacy, probably because of the need to cope with the emotions and conflicts activated by daily contact with adolescent problems [48,49,50].

It is interesting to underline that during the pandemic phase, the experienced teachers seemed to have a greater capacity for mediation, planning, and non-verbal communication. Older teachers, on the other hand, tended to show good ability for problem management and skill planning; executive and affective leadership, which is useful for dealing with the change caused by the pandemic; and a reduced perception of instructional control. The literature underlined that within the school context, the perception of lower instructional control could determine the presence of affective leadership among teachers, because it facilitates their sense of empowerment at work through participative and attuning practices [51,52]. Furthermore, during COVID-19 the grade in school seemed to influence the teachers’ ability to adapt to new situations—which appeared to be greater for middle school teachers—and the ability to plan work and use pre-verbal communication, which was higher in primary school teachers.

Regarding burnout, in the pandemic phase, younger teachers with experience showed high levels of personal satisfaction, probably because their status as young teachers belonging to the generation of “digital natives” guaranteed that they had developed sophisticated digital skills. Furthermore, primary school teachers tended to show an elevated level of burnout and, above all, depersonalization, which increased in the presence of pupils with disabilities; this was probably due to the teacher’s difficulty in involving pupils with disabilities in distance learning.

In reference to the measurement of teaching practices, during the pandemic, younger teachers with elevated experience tended to use adaptive socio-emotional and didactic strategies; furthermore, teachers who worked in the primary school tended to use practices characterized by adaptive didactic strategies and simpler language, which captured the attention of students in online teaching.

In reference to self-efficacy during the pandemic, teachers with reduced efficacy in instructional strategies and in students’ engagement taught in a numerous class in the primary school with disabled students. The data confirm the self-efficacy theory [8], which explains the role of self-efficacy in influencing behavior when facing challenges.

Confirming the first research hypothesis (H1), during the pandemic, teachers seemed to manifest a lower level of instructional competence than teachers assessed during the pre-pandemic phase. The absence of the influence of the communicative-relational dimension confirmed that the current teaching method lacks the non-verbal dimension, above all during the online teaching, in which interactions and communicative dynamics are reduced.

Confirming the second hypothesis (H2), among independent variables only elevated experience seemed to be predictive of emotional, socio-relational, and didactic competences before the COVID-19 pandemic.

The third research hypothesis was confirmed (H3), because the results show that the general level of burnout increased and personal accomplishment was reduced during the pandemic. This probably happened because teachers initially lost the ability to manage behavior in physical proximity, and experienced uncertainty about ways of managing behavior while online due to distance.

Confirming the fourth hypothesis (H4), elevated personal accomplishment was predictive of the emotional, socio-relational, and didactic competences before and during the pandemic; during Time 2, the depersonalization was a predictor of the communicative-relational and didactic dimensions. These data are confirmed by the literature, which states that greater personal satisfaction determines the presence of elevated socio-relational resources of the teacher, which is useful to cope with difficult interactions with parents and colleagues [26,35]. In addition, during the pandemic, elevated experience seemed to predict the use of the emotional and communicative-relational strategies; furthermore, teaching in a primary school with a reduced number of disabled students predicted the use of adaptive emotional strategies. In fact, to confirm the literature, an elevated experience of the teachers determined a greater ability to manage the classroom demand, the emotional climate within the school, the teacher–student interactions, and the imbalance between these demands and the available resources [9,53].

Confirming the last hypothesis (H5), the teachers’ self-efficacy could be considered a mediator of the relationship between personal accomplishment and their emotional competence during the pandemic. The data confirm Bandura’s self-efficacy theory, because teachers’ efficacy affected their motivation to organize and execute actions required to successfully accomplish a specific teaching task in a particular context; hence, teachers’ efficacy partly determines how teaching and learning activities are structured in their classrooms [8,54]. The teaching process is, therefore, a set of organizational strategies aimed at promoting in students the ability to learn and organize the contents of knowledge in an autonomous and meaningful way, so that the students are able to perform the various cognitive tasks that they intend to tackle.

The study presents some limitations, such as the use of a cross-sectional measurement that does not allow all causal relationships proposed in the theoretical framework to be measured.

Furthermore, there are some frailties regarding one measure used. In particular, three sub-scales in the ECAD-EP questionnaire presented a Cronbach’s alpha under 0.60, although the macro areas reported good estimates of internal validity.

A further limitation of the study is the use of convenience sampling methods for data collection: Although cross-sectional convenience samples may prove useful in exploring theoretical models, such as the one identified in the present study, caution should be exercised while generalizing the results beyond the current research.

Finally, because of the COVID-19 pandemic, the data collection in Time 2 was conducted with an online modality, which, compared to the face-to-face modality (used in Time 1), may have presented a lower accuracy.

Future research could analyze the impact of COVID-19 on other levels of functioning in the educational context.

## 6. Conclusions

Teachers help build a relationship with the students, from which configurations emerge that the class group reads and gives meaning. This mutual relationship presupposes that there is a common commitment to personal growth, or of a local community, as well as of an entire society. The teacher, as an educator, has the duty to interpret and give answers to the most disparate questions and should possess the following characteristics: willingness to change; the ability to define learning objectives and construct and re-construct those objectives; the ability to provide a framework, design training material, and present innovation; and the ability to increase their ability to solve problems, verifying the quality of learning. These characteristics are influenced by self-perceived instructional competence and self-efficacy.

In the current pandemic context, teachers with a high level of perceived effectiveness may experience different effectiveness given the demands of online learning, the lack of face-to-face contact with students, and the need to balance their own home life demands with simultaneous teaching requests. For example, some teachers may have an initial decrease in effectiveness in light of new needs, but their effectiveness may recover over time as they learn to adapt to the new situation or take advantage of the external resources available to them.

Present research has shown that although teachers can learn and acquire new good practices in distance learning [55,56], the school remains the environment that allows responsible teachers to provide structured learning opportunities to students [57]. Distance learning of any kind cannot therefore be considered an adequate replacement tool to deal with the pandemic situation, and above all it cannot replace face-to-face learning because the school is a place of social learning among students and a place in which to master important age-specific development tasks [58,59,60,61]. Finally, the present research has pointed out that due to the current state of education and the pandemic, teachers are facing new demands and showing high levels of burnout that are determining new educational requirements and, above all, different instructional competence. The current results are understandable. For this reason, teachers need support during this unprecedented period. Interventions could include educational, technological, or emotional support.

The information obtained from the teachers involved in the research can identify some critical issues of work in school contexts and give them an order of priority, on the basis of which improvement actions could be planned.

## Figures and Tables

**Table 1:** The estimates of the internal validity of the ECAD-EP.

**Factors**	**Variables**	**Cronbach’s Alpha**	**Cronbach’s Alpha in the Study (Phase I)**	**Cronbach’s Alpha in the Study (Phase II)**
**Socio-emotional factor**	Coexistence	0.78	0.72	0.68
	Empathy	0.74	0.69	0.67
	Communicative adaptation	0.63	0.55	0.53
	Communicative sensitivity	0.62	0.72	0.64
	Mediation	0.56	0.75	0.73
	Affective bonding	0.54	0.39	0.32
	Group dynamization	0.53	0.73	0.67
	Self-efficacy	0.51	0.66	0.65
**Communicative-relational factor**	Nonverbal communication	0.76	0.67	0.65
	Assertiveness	0.69	0.55	0.51
	Executive leadership	0.62	0.52	0.53
	Conflict resolution	0.55	0.63	0.71
	Paraverbal communication	0.46	0.35	0.41
	Affective leadership	0.46	0.38	0.46
**Didactic factor**	Instructional control	0.80	0.72	0.85
	Planning	0.71	0.65	0.82
	Adaptation	0.55	0.69	0.83

**Table 2:** *T* test for independent group: ECAD-EP (Time 1 and Time 2).

**Measures**	**Time 1**	**Time 2**	**Levene Test**		**Student’s Test**			
	**M ± S.D**	**M ± S.D**	**F**	***p*-Value**	**T**	**Df**	***p***	**Adjusted *p***
Coexistence	4.06 ± 0.47	3.79 ± 0.51	3.56	0.05	7.35		<0.001	<0.001
Empathy	4.37 ± 0.60	3.17 ± 0.44	39.454	0.000	30.267	716	<0.001	<0.001
Communication adaptation	4.51 ± 0.48	4.32 ± 0.59	14.809	0.000	4.738	716	0.001	<0.001
Communication sensitivity	4.77 ± 0.43	4.64 ± 0.60	58.211	0.000	3.261	716	0.423	0.667
Mediation	4.42 ± 0.50	4.39 ± 0.50	0.065	0.799	0.802	716	<0.001	<0.001
Affective bonding	4.18 ± 0.63	3.78 ± 0.58	2.443	0.118	8.878	716	<0.001	<0.001
Group dynamization	4.19 ± 0.52	4.11 ± 0.52	0.006	0.938	8.913	716	0.025	0.049
Self-efficacy	4.46 ± 0.43	4.36 ± 0.44	0.470	0.493	2.248	716	0.002	0.004
Nonverbal communication	4.05 ± 0.62	4.00 ± 0.61	0.678	0.411	3.173	716	0.221	0.393
Assertiveness	4.29 ± 0.62	4.23 ± 0.62	0.005	0.942	1.225	716	0.148	0.274
Executive leadership	4.45 ± 0.56	4.34 ± 0.61	8.073	0.005	1.449	716	0.012	0.024
Conflict resolution	4.45 ± 0.50	4.32 ± 0.55	6.453	0.011	2.524	716	0.002	0.004
Paraverbal communication	4.09 ± 0.67	3.66 ± 0.68	0.532	0.466	3.141	716	<0.001	0.002
Affective leadership	4.20 ± 0.67	4.03 ± 0.76	1.968	0.161	8.510	716	0.002	0.004
Instructional control	4.50 ± 0.55	4.19 ± 0.72	25.223	0.000	3.102	716	<0.001	<0.001
Planning	4.46 ± 0.47	4.18 ± 0.66	58.826	0.000	6.485	716	<0.001	<0.001
Adaptation	4.38 ± 0.64	3.77 ± 0.91	24.585	0.000	6.499	716	<0.001	<0.001
Factor A	4.37 ± 0.36	4.07 ± 0.36	0.004	0.947	10.508	716	<0.001	<0.001
Fctor B	4.26 ± 0.43	4.10 ± 0.47	1.706	0.192	11.177	716	<0.001	<0.001
Factor C	4.45 ± 0.46	4.05 ± 0.52	1.567	0.211	4.728	716	<0.001	<0.001

*Note:* Time 1 = pre-pandemic phase; Time 2 = pandemic phase; *Abbreviation*: Adjusted *p* = Bonferroni adjusted *p*-values.

**Table 3:** Multivariate two-step hierarchical modeling of burnout on the didactic factor (pre-pandemic phase).

**Model**	**Variables**	**B**	**SE**	**Beta**	**T**	***p***
1	Costant or intercept	4.523	0.234		19.338	<0.001
	Age	−0.002	0.004	−0.033	−0.337	0.736
	Years of experience	0.009	0.004	0.225	2.257	0.025
	Grade	0.081	0.056	0.074	1.445	0.149
	Role	−0.070	0.030	−0.128	−2.339	0.020
	Number of students	−0.009	0.006	0.072	−1.419	0.157
	Disabled students	0.022	0.057	0.020	0.384	0.701
2	Costant or intercept	3.584	0.251		14.304	<0.001
	Age	−0.002	0.004	−0.052	−0.580	0.562
	Years of experience	0.007	0.004	0.182	2.006	0.046
	Grade	0.072	0.051	0.066	1.421	0.156
	Role	−0.025	0.028	−0.046	−0.908	0.364
	Number of students	−0.007	0.006	−0.056	−1.208	0.228
	Disabled students	0.034	0.052	0.031	0.644	0.520
	Emotional exhaustion	−0.008	0.104	−0.020	−0.078	0.938
	Depersonalization	−0.079	0.063	−0.125	−1.247	0.213
	Personal accomplishment	0.235	0.080	0.427	2.938	0.004
	Total level of burnout	−0.020	0.252	−0.025	−0.078	0.938

*Abbreviations*: SE = standard error; β = beta standardized coefficients.

**Table 4:** Multivariate two-step hierarchical modeling of burnout on the socio-emotional factors (pandemic phase).

**Model**	**Variables**	**B**	**SE**	**Beta**	**T**	***p***
1	Costant or intercept	3.810	0.215		17.743	<0.001
	Age	0.004	0.003	0.107	1.145	0.253
	Years of experience	0.006	0.003	0.194	2.048	0.041
	Grade	−0.007	0.052	−0.007	−0.128	0.898
	Role	−0.049	0.028	−0.094	−1.733	0.084
	Number of students	−0.002	0.005	−0.023	−0.440	0.660
	Disabled students	0.081	0.063	0.067	1.279	0.202
2	Costant or intercept	2.912	0.212		13.708	<0.001
	Age	6.492 × 10^−5^	0.003	0.002	0.023	0.982
	Years of experience	0.007	0.003	0.222	2.687	0.008
	Grade	−0.010	0.046	−0.010	−0.220	0.826
	Role	−0.009	0.025	−0.017	−0.350	0.727
	Number of students	−0.002	0.005	−0.015	−0.332	0.740
	Disabled students	0.150	0.055	−0.124	−2.710	0.007
	Emotional exhaustion	−0.029	0.016	−0.090	−1.785	0.045
	Depersonalization	−0.026	0.021	−0.062	−1.233	0.219
	Personal accomplishment	0.182	0.019	0.453	9.567	<0.001

*Abbreviations*: SE = standard error; β = beta standardized coefficients.

**Table 5:** Multivariate two-step hierarchical modeling of burnout on the didactic factor (pandemic phase).

**Model**	**Variables**	**B**	**SE**	**Beta**	**T**	***p***
1	Costant or intercept	3.686	0.316		11.675	<0.001
	Age	0.000	0.005	0.005	0.051	0.959
	Years of experience	0.008	0.004	0.169	1.744	0.082
	Grade	0.129	0.077	0.090	1.673	0.095
	Role	−0.023	0.042	−0.031	−0.555	0.580
	Number of students	−0.015	0.008	−0.102	−1.889	0.050
	Disabled students	0.178	0.093	−0.102	−1.913	0.047
2	Costant or intercept	2.586	0.333		7.760	<0.001
	Age	−0.005	0.004	−0.107	−1.200	0.231
	Years of experience	0.010	0.004	0.225	2.501	0.013
	Grade	0.115	0.072	0.080	1.612	0.108
	Role	−0.015	0.039	−0.020	−0.377	0.706
	Number of students	−0.013	0.007	−0.092	−1.844	0.066
	Disabled students	0.203	0.087	0.116	2.336	0.020
	Emotional exhaustion	0.017	0.026	0.035	0.646	0.519
	Depersonalization	0.087	0.034	0.142	2.593	0.010
	Personal accomplishment	0.219	0.030	0.378	7.326	<0.001

*Abbreviations*: SE = standard error; β = beta standardized coefficients.

## Data Availability

Datasets analyzed in this study can be found by contacting the corresponding author.
